# Core decompression vs. allogenic non-vascularized bone grafting in patients with osteonecrosis of the femoral head

**DOI:** 10.3389/fsurg.2023.1219835

**Published:** 2023-08-29

**Authors:** Jin Mei, Zai-ping Jiang, Li-li Pang, Yingtao Huang, Yu Gong, Jun Zhu, Ling-wei Zhang

**Affiliations:** ^1^ Yiyang First Traditional Chinese Medicine Hospital, Yiyang, China; ^2^ Hospital of Chengdu University of Traditional Chinese Medicine, Chengdu, China; ^3^ Hunan Provincial People's Hospital, Changsha, China; ^4^ Hunan University of Traditional Chinese Medicine, Changsha, China; ^5^ Sichuan Provincial People's Hospital, Chengdu, China

**Keywords:** osteonecrosis of the femoral head, non-vascularized bone grafting, core decompression, hip-preserving procedure, hip

## Abstract

**Background:**

Core decompression and allogenic non-vascularized bone grafting are used in the early stage of osteonecrosis of the femoral head for a period. Since the comparison of the core decompression and allogenic non-vascularized bone grafting are less reported, the purpose of our study was to investigate the difference of two procedures in patients with the osteonecrosis of the femoral head.

**Methods:**

Between January 2018 and January 2019, 59 patients (64 hips) were divided into core decompression group and non-vascularized bone grafting group according to their procedures. The primary outcomes are visual analog score (VAS) and Harris hip score. Survivorship was analyzed with the collapse of the femoral head or conversion to total hip arthroplasty (THA) as the endpoint.

**Results:**

At the final follow-up, two hips underwent THA in the core decompression group and three hips in the allogenic non-vascularized bone grafting group. The radiographic survival rates were 76.9% and 77.3%, respectively, in both groups. The VAS of the core decompression group was 6.08 ± 1.164 and 3.30 ± 1.431 before and 2 years after operation (*P* < 0.05), respectively. The VAS of the allogenic non-vascularized bone grafting group was 6.00 ± 1.209 and 3.15 ± 1.537 before and 2 years after operation (*P* < 0.05), respectively. The Harris hip score of the core decompression group was 52.49 ± 6.496 before operation, and 2 years after operation, it increased by 81.14 ± 8.548 (*P* < 0.05); The Harris hip score of allogenic the non-vascularized bone grafting group was 53.56 ± 5.925 and 81.33 ± 7.243 before and 2 years after operation (*P* < 0.05), respectively. In the core decompression group, body mass index (BMI) >25 kg/m^2^ was correlated with the collapse of femoral head or conversion to THA [*P* < 0.05; 95% confidence interval (CI), 0.006–1.334], and Association Research Circulation Osseous (ARCO) III was correlated with the collapse of femoral head or conversion to THA (*P* < 0.05; 95% CI, 2.514–809.650). In the allogenic non-vascularized bone grafting group, age, BMI, and ARCO stage were significantly associated with the collapse of femoral head or conversion to THA (*P* > 0.05).

**Conclusion:**

The clinical survival rate of the femoral head in the core decompression group was slightly better than that in the allogenic non-vascularized bone grafting group. There was no significant difference in the radiographic survival rate of the femoral head between the two groups. Both groups can alleviate pain and improve functional of patients, but there was no significant difference in the degree of improvement. In the core decompression group, BMI >25 kg/m^2^ and ARCO III correlated with the collapse of femoral head or conversion to THA. In the allogenic non-vascularized bone grafting group, no association was found between age, BMI, and ARCO stage and the collapse of femoral head or conversion to THA.

**Level of evidence:**

III.

## Background

Osteonecrosis of the femoral head (ONFH) is a common disease and often occurs in alcoholics or those receiving hormone therapy (1, 2). Hip arthroplasty is the main treatment with good outcomes (3), but hip-preserving techniques are also important for young patients with osteonecrosis of the femoral head (4). With the development of diagnostic technology and the improvement of patients’ health awareness (5), the number of young patients with avascular necrosis of the femoral head has increased. Current hip-preserving procedures include core decompression, non-vascularized or vascularized bone grafting, and osteotomy (6–9).

Core decompression is a safe, effective, and less invasive surgery. At the same time, it is also the most cost-effective procedure in hip-preventing techniques (10). It can be divided into multiple drilling techniques and conventional core decompression. Core decompression can reduce the pressure in the femoral head, remove the femoral head necrosis tissue, stimulate the formation of blood vessels, promote the formation of new bone, and delay the process of avascular necrosis of the femoral head (11). Core decompression combined with other therapies also procures good outcomes, including autologous bone marrow stem cells (12) or bone morphogenetic protein (13). Core decompression is a new way for treating osteonecrosis of the femoral head.

Allogenic non-vascularized bone grafting was first proposed by Phemister in 1949 (14). It can provide good support that is beneficial to the repair and reconstruction of subchondral bone, thereby delaying the progress of avascular necrosis of the femoral head and the time to hip arthroplasty. The most commonly used surgical methods include the “Phemister technique,” “lightbulb,” and “trapdoor” (15).

Core decompression and allogenic non-vascularized bone grafting were used in the early stage of osteonecrosis of the femoral head frequently. Both procedures show good results. However, which procedure has higher priority still needs to be discussed under different circumstances. This study aims to explore the difference between core decompression and allogenic non-vascularized bone grafting in hip preservation.

## Materials and methods

We retrospectively reviewed the data of all patients at the Affiliated Hospital of Chengdu University of Traditional Chinese Medicine during the period between January 2018 and January 2019. All methods were carried out following relevant guidelines and regulations. Patients who met the following criteria were included: (1) those who underwent either core decompression or allogenic non-vascularized bone grafting for ONFH; (2) the staging of avascular necrosis of the femoral head is between Association Research Circulation Osseous (ARCO) I and II (pre-collapsed stage) or ARCO III (early collapsed stage) (16); (3) The age of the patient is 18–70 years. Patients who met the following criteria were excluded: (1) patients with an advanced stage of osteonecrosis of the femoral head (worse than ARCO IIIA or collapsed); (2) broken skin or infection to the hip; (3) patients who received other types of hip-preserving procedures.

**Figure 1:**
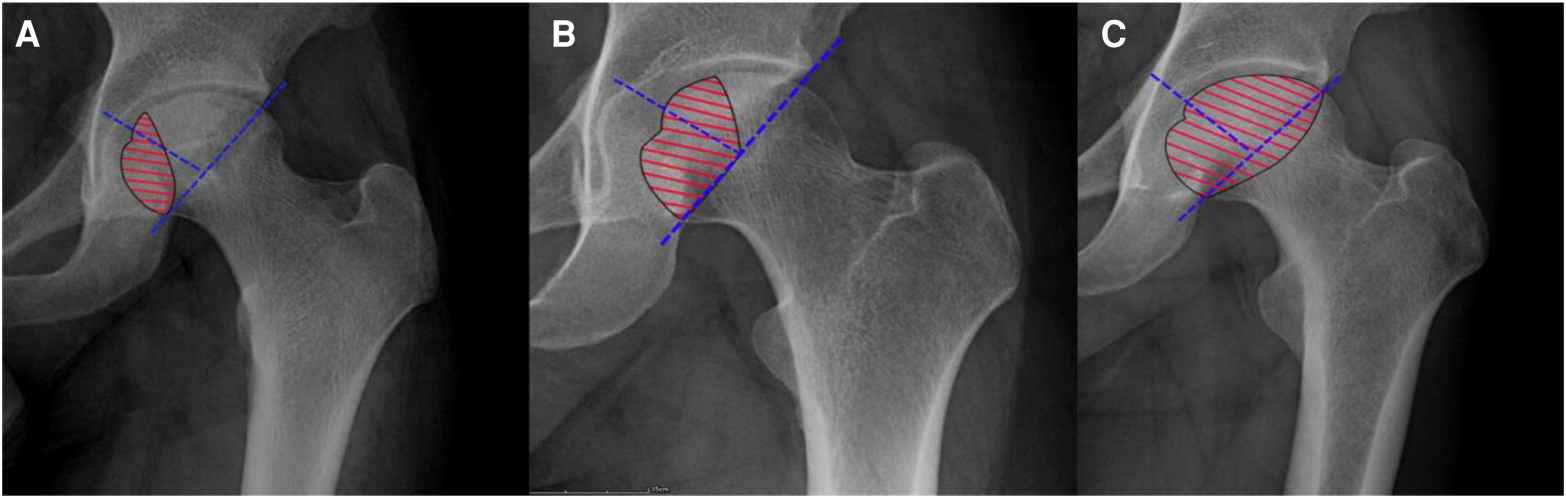
(**A**) A medial-type lesion occupied the medial one-third of the femoral head not involving the weight-bearing portion. (**B**) A central-type lesion occupied the medial two-thirds or less of the weight-bearing portion. (**C**) A lateral-type lesion occupied more than the medial two-thirds of the weight-bearing portion.

All patients were diagnosed with ONFH on anteroposterior radiographs or magnetic resonance imaging (MRI). Before surgery, all patients were informed of the advantages and disadvantages of both surgical procedures, and the patients chose the surgical procedure by themselves. They underwent laboratory evaluation including routine blood tests, liver and kidney function, and electrolytes. The baseline variables of patients including age, gender, etiology, ARCO stage, lesion size, location of the lesion, and body mass index (BMI) were recorded. Lesion size can be divided into three types according to the percent of lesion size on the femoral head. The involvement of lesion size on the femoral head less than 15% was defined as small type, 15%–30% was medium type, and more than 30% was the large type. The estimation of lesion size is based on a mid-coronal section of the femoral head and the involved layers on computed tomography (CT) or MRI (17). The location of the lesion was classified as medial, central, and lateral (Figure 1). The visual analog score (VAS) and Harris hip score (HHS) were also recorded.

## Surgical procedures

### Core decompression

All procedures are completed by a senior surgeon with the same surgical technique: patients were placed in the supine position on an operating table after anesthesia. The skin was cut longitudinally about 2–3 cm from the place 2 cm below the greater trochanter of the femur, and with the help of the C-arm, a guide needle was placed at a certain position to the necrosis area of the femoral head. Several passages were made to stimulate revascularization of the entire necrotic part of the femoral head.

### Allogenic non-vascularized bone grafting

**Figure 2:**
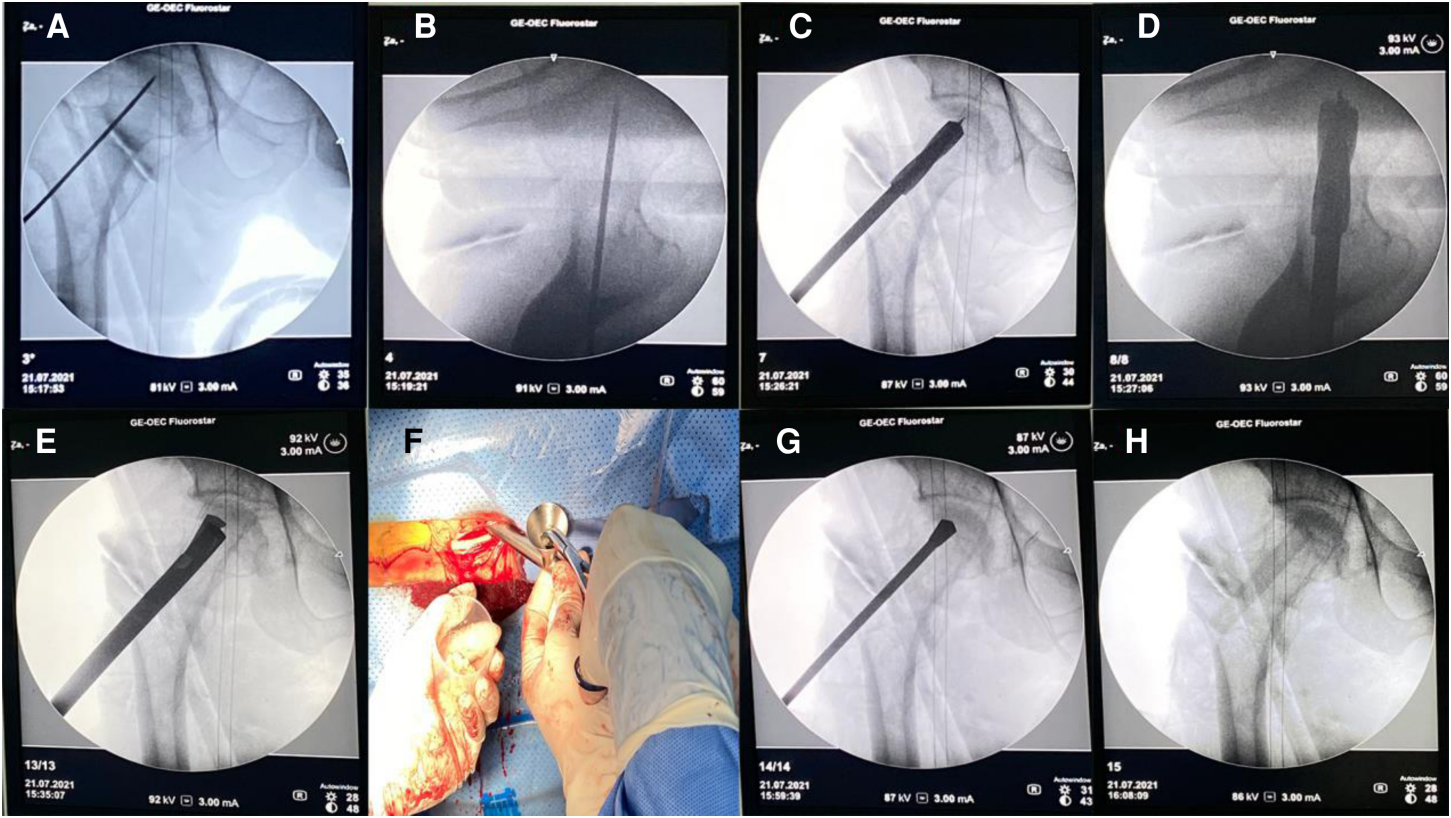
(**A**) Kirschner wire positioning (anterior–posterior position). (**B**) Kirschner wire positioning (lateral position). (**C,D**) Empty the drill and build a decompression tunnel. (**E**) Scrape the necrotic bone. (**F**) implant the allogenic cancellous bone. (**G**) Properly press and compact the bone. (**H**) Allogeneic bone is in place.

**Figure 3:**
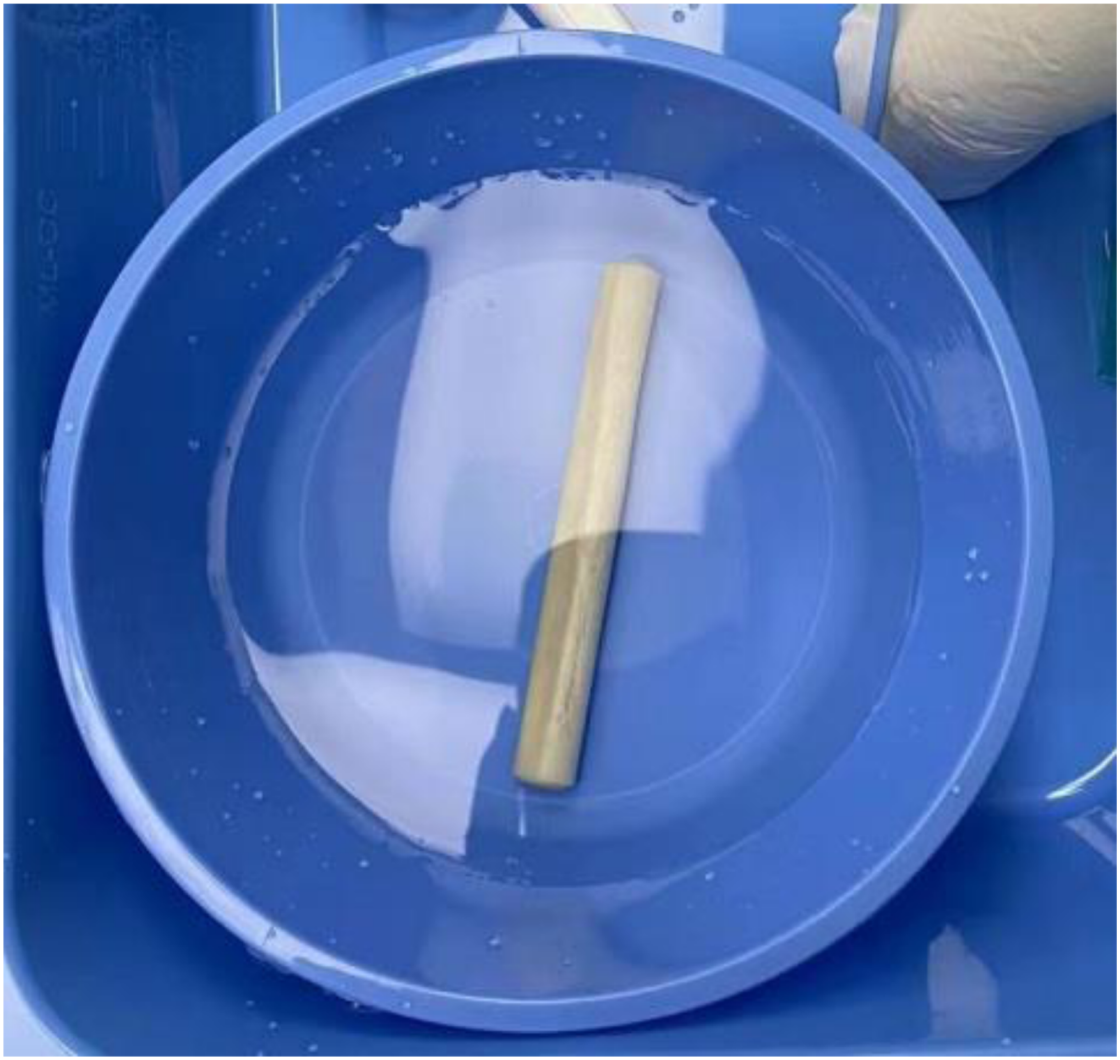
Allogeneic bone (Shanxi Orui Biomaterials Co., Ltd.). Length 100 mm.

All procedures were completed by a senior surgeon with the same surgical technique (Figure 2): patients were placed in the supine position on an operating table after anesthesia. The skin was cut longitudinally about 2–3 cm from the place 2 cm below the greater trochanter of the femur, and with the help of the C-arm, a guide needle was placed at a certain position to the necrosis area of the femoral head. The Kirschner wire can be seen in the center and was advanced to a position 5 mm from the subchondral bone of the femoral head under the C-arm guidance. A trephine with K-wire was inserted to remove the bone tissue in the necrotic area. The allogeneic cancellous bone is implanted into the necrotic area and allogeneic non-vascularized bone (Figure 3) was implanted finally.

All patients were educated to keep their operative limb unweighted with the crutches for 3 months. For the next 3 months, partial weight-bearing was allowed. Patients were then advised to start walking without crutches. Strenuous exercise was not recommended.

### Outcomes

**Figure 4:**
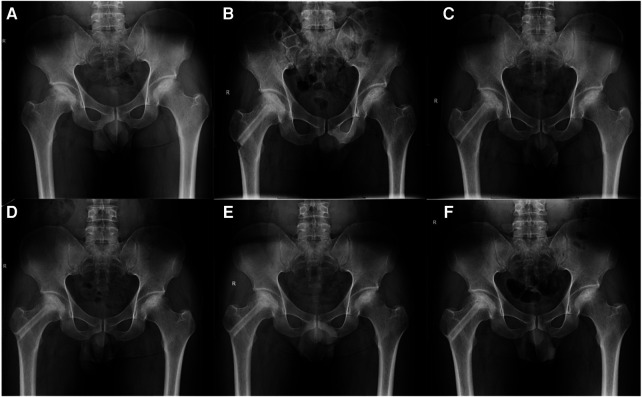
A 39-year-old male patient with alcohol-related ONFH on the right hip. (**A**) Pre-operative radiograph. (**B**) Non-vascularized bone was grafted at post-operative radiograph. (**C**–**E**) The necrotic area is being repaired by the support of allograft at 3, 6, and 12 months post-operatively. (**F**) Follow-up radiography at 33 months post-operatively showing no radiography progress on his right hip.

Conversion to total hip replacement is defined as the endpoint of clinical outcome, and the occurrence of the collapse of femoral head is defined as the endpoint of radiographic outcome. The procedure is defined as clinical success if the patients preserved the native femoral head. The collapse of the femoral head is defined as radiography failure (Figure 4). VAS and HHS were recorded pre-operatively and 6 months, 1 year, and 2 years after the procedure. The Harris hip score of fewer than 70 points is ranked as poor, 70–79 points is fair, 80–89 points is good, and more than 90 points is excellent.

## Statistical analysis

A normality test was performed for numerical variables if all groups satisfied the normal distribution and the variances between the two groups are equal; mean ± standard deviation (SD) and two independent sample *t*-test were used to describe the comparison between groups. The number of cases (percentage) was used for categorical variables, and *χ*^2^ test was performed between groups. Paired *t*-test was performed to compare pre-operative VAS and HHS with those after procedure. We calculated two sets of power values in VAS and Harris hip scores using sample size, effect size, and significant level in R studio. Kaplan–Meier survival curves with the endpoint of collapse or conversion to total hip arthroplasty (THA) and log-rank analysis were used for statistical differences in survival rate. A binary logistic regression test was used to analyze risk factors that lead to collapse or conversion to THA. *P* < 0.05 was considered significant for statistical comparisons. All statistical comparisons were performed by the Statistical Package for Social Sciences (SPSS) (version 22).

## Results

Between January 2018 and January 2019, 59 patients (64 hips) were divided into the core decompression group and the allogenic non-vascularized bone grafting group according to their procedures: 37 hips in the core decompression group and 27 hips in the allogenic non-vascularized bone grafting group. In the core decompression group, 24 hips were ARCO stage II and 13 were stage III. Twenty hips were ARCO stage II and seven were stage III in the allogenic non-vascularized bone grafting group. The mean follow-ups were 39.43 ± 3.8 months (31–48 months) and 39.48 ± 5.8 months (26–48 months), respectively. The patients were aged 22–70 years (mean, 47.5 ± 11.1) and 27–66 years (mean, 40.6 ± 12.0) in the core decompression group and the allogenic non-vascularized bone grafting group, respectively. Baseline variables of patients including age, sex, height, weight, BMI, and mean follow-up period were collected (Table 1). Baseline variables of disease including etiology, ARCO classification, lesion size, and location of lesion were also collected. There were no significant differences between patients in the core decompression group and those in the allogenic non-vascularized bone grafting group in terms of the baseline variables. The percentage of follow-up completed at 2 years in the core decompression group was 94.6% and in the allogenic non-vascularized bone grafting group was 92.6%. The percentage of follow-up completed at 2 years in both groups was 93.8%.

**Table 1:** Baseline variables of patients in core decompression and non-vascularized allogeneic fibula grafting groups^a^.

Variables	Core decompression	Non-vascularized bone grafting	*P*-value
	(*N* = 37)	(*N* = 27)	
Age	47.5 + 11.1 (22–70)	40.6 ± 12.0 (27–66)	0.113
Male:female	31:6	21:6	0.385
Mean follow-up period (months)	39.43 ± 3.8 (31–48)	39.48 ± 5.8 (26–48)	0.967
Etiology (no. of hips)			0.094
Alcohol use	26 (73.1%)	14 (51.9%)	
Idiopathic	1 (3.8%)	5 (18.5%)	
Steroid use	10 (23.1%)	7 (25.9%)	
Traumatic	0 (0%)	1 (3.7%)	
ARCO classification			0.3.06
ARCO II	24 (64.9%)	20 (74.1%)	
ARCO III	13 (35.1%)	7 (25.9%)	
Lesion size			0.995
Small	8 (21.6%)	6 (22.2%)	
Medium	10 (27.0%)	7 (26.0%)	
Large	19 (51.4%)	14 (51.8%)	
Location of lesion			0.767
Medial	6 (16.2%)	6 (22.2%)	
Central	13 (35.1%)	10 (37.0%)	
Lateral	18 (48.7%)	11 (40.8%)	
Height (cm)	165.92 ± 6.95 (155–180)	166.81 ± 7.48 (150–176)	0.624
Weight (kg)	64.32 ± 10.67 (44–86)	65.56 ± 11.04 (44–88)	0.655
BMI (kg/m^2^)	23.27 ± 3.0 (18.1–28.4)	23.31 ± 2.82.9 (18.1–28.1)	0.912

^a^ Values are presented as mean ± SD (range) or number of cases (percentage).

The primary outcomes included VAS and HHS were recorded (Table 2). The mean pre-operative VAS was 5.70 points (range, 4–8 points) in the core decompression group. The mean pre-operative VAS was 5.86 points (range, 4–9 points) in the allogenic non-vascularized bone grafting group. Two sets of baseline data were comparable (*P* > 0.05). The last follow-up score was 3.61 ± 1.53 and 3.55 ± 1.53 points in the core decompression group and the allogenic non-vascularized bone grafting group, respectively. The difference is significant compared with baseline scores (*P* < 0.05). The mean pre-operative HHS were 51.4 points (range, 42–61 points) and 52.8 points (range, 41–63 points) in the core decompression group and the allogenic non-vascularized bone grafting group, respectively. Two sets of baseline data were comparable (*P* > 0.05). The last follow-up score was 76.5 ± 12.9 and 79.2 ± 10.0 points in the core decompression group and the allogenic non-vascularized bone grafting group, respectively. The difference was significant compared with baseline scores (*P* < 0.05), and power was <0.8 in power analysis.

**Table 2:** Primary outcomes of patients in core decompression group and non-vascularized bone grafting groups^a^.

	Core decompression	Non-vascularized bone grafting group	Between-group difference
VAS			
Baseline	5.70 ± 1.18 (4–8)	5.86 ± 1.21 (4–9)	0.16 (−0.79 to 0.60)
6 months	4.27 ± 1.12^b^	4.64 ± 0.95^b^	0.37 (−0.98 to 0.24)
1 year	3.89 ± 0.95^b^	3.96 ± 1.13^b^	0.07 (−0.68 to 0.54)
2 years	3.61 ± 1.53^b^	3.55 ± 1.53^b^	0.06 (−0.82 to 0.96)
HHS			
Baseline	51.4 ± 6.5	52.8 ± 5.8	1.4 (−5.0 to 2.2)
6 months	61.8 ± 9.5^b^	62.6 ± 6.7^b^	0.8 (−5.7 to 4.0)
1 year	69.3 ± 11.5^b^	71.2 ± 8.6^b^	1.9 (−8.0 to 4.0)
2 years	76.5 ± 12.9^b^	79.2 ± 10.0^b^	2.7 (−9.6 to 4.1)

^a^ Values are presented as mean ± SD (95% CI).

^b^ Significantly different compared with baseline score by using paired Student's *t*-test within same group (*P* < 0.05).

**Figure 5:**
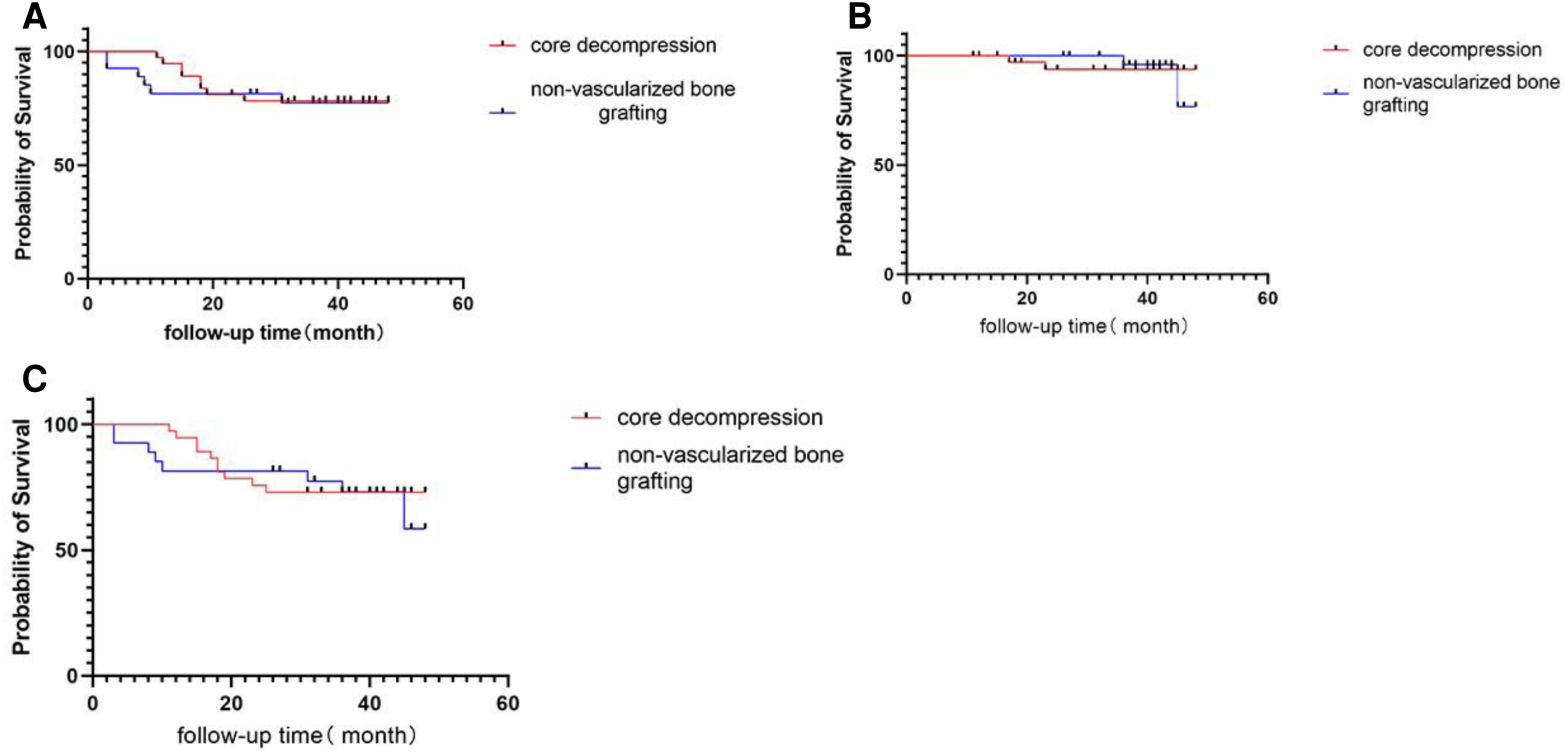
(**A**) Kaplan–Meier survival curve of radiographic failure with collapse as the endpoint. (**B**) Kaplan–Meier survival curve of clinical failure with conversion to THA as the endpoint. (**C**) Kaplan–Meier survival curve of the overall survival rate.

Occurrences where patients who had clinical and radiographic failures (defined as conversion to total hip replacement and collapse of femoral head) were recorded. The radiographic survival rate is 76.9% in the core decompression group at the last follow-up time, and in the allogenic non-vascularized bone grafting group, the radiographic survival rate is 77.3% (Figure 5A). Occurrence of THA in patients after the procedure was defined as clinical failure. Two hips underwent THA after core decompression and the clinical survival rate was 94.6%. Two hips underwent THA after allogenic non-vascularized bone grafting and the clinical survival rate was 92.6% (Figure 5B). The overall survival of core decompression groups and allogenic non-vascularized bone grafting is 73.0% and 63.0% (Figure 5C).

A binary logistic regression test (Table 3) showed that lateral-type lesion [*P* = 0.03; 95% confidence interval (CI), 0.765–2.114], BMI >25 kg/m^2^ (*P* = 0.35; 95% CI, 0.999–2.131), and ARCO III stage (*P* = 0.28; 95% CI, 1.254–58.752) were independent risk factors for the collapse of femoral head and conversion to THA in the core decompression group. In the allogenic non-vascularized bone grafting group, ARCO III stage (*P* = 0.02; 95% CI, 1.351–49.344) and BMI >25 kg/m^2^ (*P* = 0.35; 95% CI, 0.999–2.131) were independent risk factors for the collapse of femoral head and conversion to THA.

**Table 3:** Risk factor analysis.

	Core decompression group^a^			Non-vascularized bone grafting group^a^		
Variable	*P*	Exp(B)	CI	*P*	Exp(B)	CI
Age	0.080	0.093	0.006–1.334	0.995	0.994	0.146–6.764
BMI (kg/m^2^)	0.009	24.461	2.188–273.405	0.382	2.180	0.380–12.506
ARCO	0.010	45.115	2.514–809.650	0.743	1.443	0.162–12.875

^a^ Hosmer–Lemeshow (HL), *P* > 0.05.

## Complications

No complications occurred in the core decompression group. However, two patients had severe complications after allogenic non-vascularized bone grafting. One patient had an intertrochanteric fracture after the procedure and the other one had an infection 28 months after the procedure.

## Discussion

This trial is the first to compare core decompression and allogenic non-vascularized bone grafting for ONFH. In our study, HHS and VAS of patients were improved at 2 years, but there was no significant difference between the core decompression and allogenic non-vascularized bone grafting groups at any time point up to 2 years. The radiographic survival rates were 76.9% and 77.3% in core decompression and allogenic non-vascularized bone grafting, respectively.

Pain and functional activity are the main concerns of patients in hip-preserving procedures because it affects the quality of daily life. In our study, core decompression and allogenic non-vascularized bone grafting reduced the pain of patients and improved their functional activities. Core decompression is mainly to reduce the pain of the patient by reducing the high pressure in the bone, and allogenic non-vascularized bone grafting reduces subchondral microfractures and relieves pain by the support of the allograft bone. However, there is no significant difference between the two groups in the degree of improvement.

Osteonecrosis of the femoral head is characterized by the death of osteoblasts and the collapse of the joint surface of the femoral head due to the interruption of the vascular supply. Pain and dysfunction at the late stage can seriously affect the patient's quality of life. Total hip replacement is still the main option for the treatment of advanced femoral head necrosis. However, younger patients may need revision considering the limited life span of the prosthesis. Although the development of prosthetic materials, such as high cross-linked polyethylene materials, metal-to-metal joints, and ceramic bone grafts, reduces osteolysis caused by polyethylene wear particles and extends the period of use of prostheses, shortcomings still exist. About 7% and 80% of patients with small and large lesions, respectively, collapse by 8 years without intervention (18). Hip-preserving treatment can relieve pain, delay the collapse of the articular surface, and delay the time to THA.

There are many treatments for osteonecrosis of the femoral head. Core decompression and allogenic non-vascularized bone grafting are hip-conserving treatments, which are suitable for the treatment of osteonecrosis of the femoral head at the early stage and the femoral head has not yet collapsed. Core decompression is a safe, effective, and less invasive surgery. It can clear dead bones, reduce pressure inside the femoral head, and promote bone tissue regeneration. Meanwhile, the combination of core decompression with orthobiologics such as bone morphogenetic protein (BMP), stem cells, and platelet-rich plasma (PRP) may achieve better results. In their meta-analysis, Wang (12) found that the combination of core decompression and autologous bone marrow stem cells has a better effect than simple core decompression. Martinot et al. (13) found that reinjection of bone marrow and/or BMP combined with core decompression can improve hip survival.

Lesion size may influence the survival rate of the femoral head in core decompression. In the hip with a small necrosis area, the failure rate reached 14%–25%, while in the femoral head with a larger necrosis area, the failure rate reached 42%–84% (4). This was also confirmed in our article. Serong et al. found that the effect of core decompression is related to age and patients older than 40 years have a higher failure rate, but there is no significant correlation with gender (19). However, we did not find this correlation in our study. Strong repair capabilities of young patients may lead to this, but they also have a higher impact on loading activities. Octavian found in a meta-analysis that 38% of the 1,134 hips that underwent core decompression had THA at an average follow-up time of 26 months (20). Core decompression combined with other therapies (bone marrow stromal cells) can also achieve good results in the early stages of femoral head necrosis (21, 22). Different procedures may result in varied outcomes. Al Omran proposed that there is no difference between traditional core decompression and multiple drilling (23). Song et al. reported that 88% of hips with small and medium lesion sizes did not have THA in a multiple drilling core decompression group (24). Multiple drilling core decompression was less invasive compared to traditional core decompression.

Compared with core decompression, allogenic non-vascularized bone grafting has better mechanical support, which may be more advantageous to prevent the collapse of the femoral head. Non-vascularized bone grafting has shown a good survival rate of hips in the literature (25, 26). Sultan et al. concluded in a review that the survival rate of non-vascularized bone grafting in hips is between 62% and 86% within an average follow-up time of 24–104 months after surgery (27). Wu et al. reported the long-term outcomes of non-vascularized bone grafting. In a study with an average follow-up time of 14 years, the clinical survival rate of the hips was 62.5% (28). Non-vascularized allogeneic fibula grafting combined with core decompression is an effective treatment method, and the success rate of that in 7 years is 81.8%. Changjun et al. holds that the biological and biomechanic factors are important (29). In our study, the clinical survival rate was 86.4% at a mean of 29.2 months in the allogenic non-vascularized bone grafting group. ARCO III stage was a risk factor in the allogenic non-vascularized bone grafting group. Advanced ARCO stage portended to unsuccessful clinical results. This was proved in many studies (4, 30). Nelson and Clark also pointed out that non-vascularized bone grafting is not recommended for patients with femoral head collapse (31). We also found that BMI >25 kg/m^2^ is an independent risk factor for the collapse of femoral head in allogenic non-vascularized bone grafting. This may provide advice to the surgeon.

There are some limitations in this study, including a small number of patients and a short follow-up time (mean, 39.43 and 39.48 months, respectively). However, the minimum 3-year follow-up period is enough to evaluate the pain and activities of patients. The design of the study is retrospective and patient selection and disease characteristics are well-defined, with a power of <0.8 in power analysis. This means there is excessive overlap between two distributions or small sample size. In addition, the clinical and radiographic failures are well defined. All the baseline data and outcomes were recorded completely in detail. This renders our results representative of the short-term outcomes of core decompression vs. allogenic non-vascularized bone grafting in the treatment of ONFH. However, long-term follow-up clinical trials are still needed.

## Conclusion

In this study, the clinical survival rate of the femoral head in the core decompression group is slightly better than that in the allogenic non-vascularized bone grafting group. There was no significant difference in the radiographic survival rate of the femoral head between the two groups. Core decompression and allogenic non-vascularized bone grafting can reduce patients' pain and improve their functional activity, but there is no significant difference in the degree of improvement. In the core decompression group, BMI >25 kg/m^2^ and ARCO III were correlated with femoral head collapse or conversion to THA. In the allogenic non-vascularized bone grafting group, no association was found between age, BMI, and ARCO staging and femoral head collapse or THA.

## Data Availability

The original contributions presented in the study are included in the article, further inquiries can be directed to the corresponding author.
